# Correlation Between Anastomotic Leakage and the Anastomotic Position of the Gastric Conduit After Esophagectomy Using ICG Fluorescence Imaging Stratification

**DOI:** 10.1002/wjs.70552

**Published:** 2026-08-30

**Authors:** Yamato Ninomiya, Kazuo Koyanagi, Rie Nakashima, Kohei Tajima, Kohei Kanamori, Mika Ogimi, Yoshiaki Shoji, Akihito Kazuno, Miho Yamamoto, Masaki Mori

**Affiliations:** ^1^ Department of Gastroenterological Surgery Tokai University School of Medicine Isehara Japan

**Keywords:** anastomotic leak, esophagectomy, fluorescence imaging, gastric conduit perfusion, indocyanine green

## Abstract

**Background:**

This study aimed to clarify the correlation between anastomotic leakage and anastomotic position after esophagectomy for esophageal cancer, stratified by gastric conduit blood flow assessed using indocyanine green (ICG) fluorescence imaging.

**Methods:**

A total of 136 patients who underwent esophagectomy with retrosternal gastric conduit reconstruction were included. After creating the gastric conduit, three points were identified: point A (anastomotic site), point B (terminal end of the gastroepiploic artery stained with ICG), and point C (terminal end of the gastric conduit wall stained with ICG). Anastomotic position was defined by the relative location of point A between points B and C, and blood flow speed before reconstruction (pre‐speed) was quantified using ICG fluorescence imaging. Associations of anastomotic position and pre‐speed with anastomotic leakage were evaluated.

**Results:**

Anastomotic leakage occurred in 22 patients (16.2%). Mean distance BA (point B to A) was 2.05 cm in the leakage group and 0.15 cm in the non‐leakage group (*p* = 0.018). Mean distance AC (point A to C) was 4.00 cm in the leakage group and 5.89 cm in the non‐leakage group (*p* = 0.016). Logistic regression revealed that BA > −0.50 cm and pre‐speed < 2.19 cm/s were independent risk factors for anastomotic leakage (odds ratio 4.67, *p* = 0.046, and odds ratio 56.16, *p* < 0.001, respectively). Among patients with delayed blood flow, the anastomotic leakage rate was significantly lower in the proximal group than in the distal group (*p* = 0.034).

**Conclusions:**

ICG fluorescence imaging provides useful information for determining an appropriate anastomotic position. Performing the anastomosis at a more proximal site within the gastric conduit may reduce the risk of anastomotic leakage.

## Graphical abstract

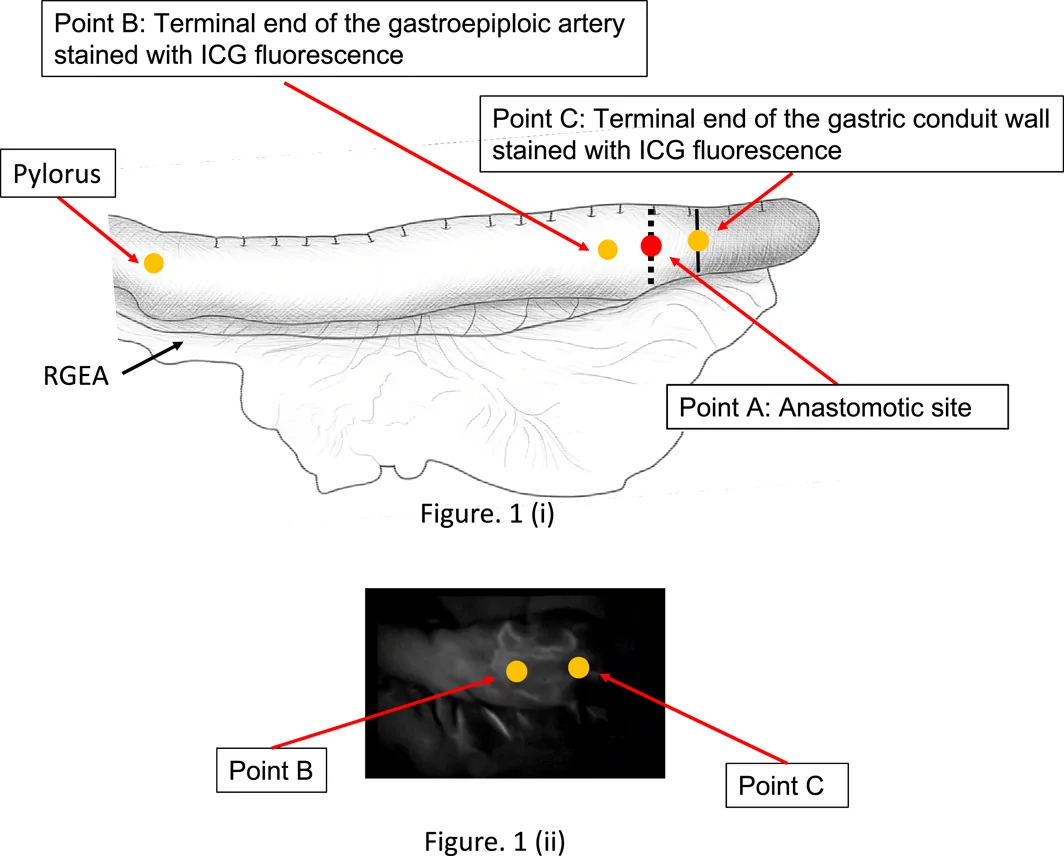

## 1 Introduction

Anastomotic leakage is a common and serious complication after esophagectomy and is associated with prolonged hospitalization and increased postoperative mortality [1, 2, 3, 4, 5]. Therefore, prevention of anastomotic leakage is essential for improving outcomes after esophagectomy. Blood flow in the gastric conduit has long been considered critical for reducing the risk of anastomotic leakage and has been extensively studied; however, no objective reports have evaluated both blood flow and the anastomotic position on the gastric conduit [6, 7, 8, 9].

In a previous study, we assessed the association between blood flow speed in the gastric conduit wall—measured using indocyanine green (ICG) fluorescence—and anastomotic leakage after esophagectomy, and demonstrated that delayed blood flow speed was a significant risk factor [10]. Patients with a non‐delayed blood flow speed had a low risk of leakage; however, leakage still occurred in some patients despite relatively fast blood flow. These findings suggested that, beyond vascular perfusion alone, other factors such as the reconstruction route and anastomotic position may influence anastomotic integrity. As an initial additional investigation, we examined the effect of the thoracic inlet space on blood flow speed and anastomotic leakage during retrosternal reconstruction [11]. We found that a narrow thoracic inlet space caused compression of the gastric conduit, delayed blood flow speed, and increased the risk of leakage.

As the next step, we focused on the anastomotic position of the gastric conduit. Because the blood supply to the gastric conduit is primarily derived from the right gastroepiploic artery, it is generally assumed that perfusion is better on the proximal side; however, this assumption has not been thoroughly investigated. We hypothesized that the anastomotic position is associated with differences in blood flow and may serve as an important determinant of leakage risk.

The aim of the present study was to clarify the relationship between anastomotic leakage and anastomotic position in the gastric conduit after esophagectomy. Using ICG fluorescence imaging, we objectively assessed blood flow and determined the anastomotic position. Patients were stratified into groups with delayed versus non‐delayed blood flow speed, and we evaluated the impact of anastomotic position on the occurrence of anastomotic leakage.

## 2 Material and Methods

### 2.1 Patients

Informed consent for surgical procedures and ICG fluorescence studies was obtained from all patients who underwent esophagectomy at Tokai University Hospital. Patients with iodine allergy were excluded. Among consecutive eligible patients diagnosed with thoracic esophageal cancer between June 2014 and February 2018, 149 patients who provided preoperative consent were enrolled. To eliminate the potential influence of differences in anastomotic technique, six patients who underwent mechanical end‐to‐side anastomosis were excluded. In addition, to eliminate the influence of reconstruction route on anastomotic leakage, only patients who underwent retrosternal route reconstruction were included. Seven patients who underwent antethoracic reconstruction were excluded: two because of intraoperative hypotension or arrhythmia during retrosternal tunnel creation and five because of chest wall deformities such as funnel chest. After these exclusions, 136 patients were included in the final analysis.

Medical histories, including hypertension, diabetes, dyslipidemia, acute myocardial infarction, angina pectoris, cerebral infarction, and cerebral hemorrhage, were recorded. Pathological factors were classified according to the Union for International Cancer Control (UICC) TNM classification, 8th edition [12].

### 2.2 Surgical Procedure and Definition of Anastomotic Leakage

Thoracoscopic resection is the standard surgical approach at our institution; however, conversion to open thoracotomy was required in 13 cases due to intrapleural adhesions [13]. Abdominal procedures were performed laparoscopically, either as hand‐assisted laparoscopic surgery (HALS) (*n* = 102) or via laparotomy (*n* = 34). The right gastroepiploic artery and vein were preserved, and a 3.5‐cm‐wide gastric conduit was created by incising the lesser curvature 5 cm from the pylorus, without preserving the omentum. Kocher mobilization was not routinely performed but was selectively added when additional conduit length was required. The gastric conduit was then pulled up through the retrosternal route and anastomosed to the cervical esophagus.

Hand‐sewn end‐to‐end, layer‐to‐layer anastomoses were performed in all patients. The mucosa was sutured with continuous 4‐0 monofilament absorbable sutures, and the seromuscular layer was sutured with interrupted 4‐0 multifilament absorbable sutures.

Anastomotic leakage was defined as a Clavien–Dindo Grade II or higher complication [14]. Esophagography and/or CT examination were performed on postoperative day 6 to evaluate leakage [15]. Additionally, clinical findings such as saliva discharge from the cervical wound were considered diagnostic of anastomotic leakage.

### 2.3 ICG Fluorescence Imaging

ICG administration and fluorescence measurements were performed before reconstruction. Imaging was conducted with the distal end of the prepared gastric conduit positioned cranially on the chest wall. Before ICG injection, the conduit length and color, pulsation of the right gastroepiploic artery, and the presence of a gastroepiploic arterial communication (arterial arcade) were visually assessed and recorded. Gastroepiploic arterial communication was defined as grossly visible continuity between the right and left gastroepiploic arteries after gastric conduit creation. This finding was assessed intraoperatively.

ICG dye (Diagnogreen; Daiichi‐Sankyo Pharmaceutical, Tokyo, Japan) was administered as a bolus at a constant rate via a central venous catheter at a standard dose of 1.25 mg. Fluorescence was observed using a near‐infrared camera system (Photodynamic Eye [PDE]; Hamamatsu Photonics K.K., Hamamatsu, Japan).

### 2.4 Assessment of Anastomotic Position Using ICG Fluorescence Imaging

For this study, the pylorus side of the gastric conduit was defined as proximal, and the apical side as distal. As shown in Figure 1, three points were defined using ICG fluorescence imaging: point A, the anastomotic site; point B, the terminal end of the gastroepiploic artery visualized by fluorescence; and point C, the terminal end of the gastric conduit wall visualized by fluorescence. The anastomotic site (point A) was generally set proximally relative to point C. The BA, AC, and BC distances were calculated as signed distances based on imaging findings (A−B, C−A, and C−B, respectively). Negative values indicated that the measured point was located proximal to the corresponding reference point.

**FIGURE 1:**
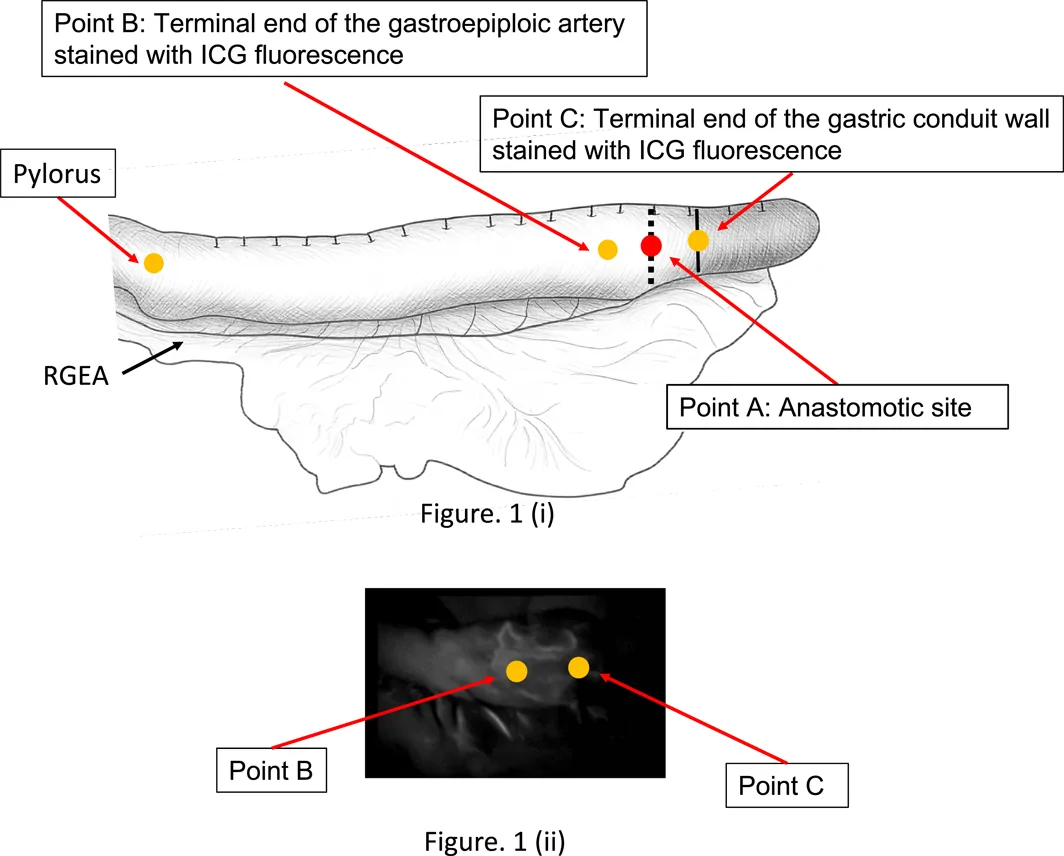
(i) Evaluation of anastomotic position of the gastric conduit and blood flow speed using ICG fluorescence imaging. (ii) The photograph shows points B and C identified by ICG fluorescence imaging. Point A: Anastomotic site; Point B: Terminal end of the gastroepiploic artery stained with ICG fluorescence; Point C: Terminal end of the gastric conduit wall stained with ICG fluorescence. The following distances (cm) were measured. Distance A: The distance from pylorus to point A. Distance B: The distance from pylorus to point B. Distance C: The distance from pylorus to point C. Distance BA: The distance from point B to point A. Distance AC: The distance from point A to point C. Distance BC: The distance from point B to point C. ICG: indocyanine green, RGEA: right gastroepiploic artery.

### 2.5 Blood Flow Speed in the Proximal and Distal Gastric Conduit

Blood flow speed assessed using ICG fluorescence imaging was measured intraoperatively and subsequently reevaluated by a single gastrointestinal surgeon using the recorded videos. Flow time was defined as the time from ICG injection to the first appearance of any detectable fluorescence at point C. The overall moving speed of ICG (pre‐speed, cm/s) was calculated using the distance from the pylorus to point C and the time required for fluorescence to reach point C [10, 11]. Point C was selected as the endpoint for pre‐speed measurement because it could be consistently identified before reconstruction, whereas point A was determined only after gastric conduit pull‐up. Throughout this manuscript, blood flow speed measured before reconstruction is referred to as “pre‐speed” to distinguish it from post‐reconstruction blood flow speed evaluated in our previous studies. Proximal pre‐speed (from the pylorus to point B) and distal pre‐speed (from point B to point C) were also calculated and compared.

### 2.6 Statistical Analysis

Clinical variables, gastric conduit measurements, and group comparisons were analyzed using Fisher's exact test for categorical variables and the Mann–Whitney *U* test for continuous variables. The Wilcoxon signed‐rank test was used to compare proximal and distal pre‐speed within the same patients. Receiver operating characteristic (ROC) curve analysis was performed to determine the cutoff values of distance BA, distance AC, and pre‐speed for anastomotic leakage. The optimal cutoff values were determined using the Youden index. For risk analysis of anastomotic leakage, multivariable logistic regression was performed including the distance BA, distance AC, and pre‐speed, which were the primary variables of interest. For pairwise comparisons of anastomotic leakage rates among the four groups, *p*‐values were adjusted using the Bonferroni correction to account for multiple comparisons. A *p*‐value < 0.05 was considered statistically significant. All analyses were performed using SPSS Statistics version 26.0 (IBM Japan, Tokyo, Japan).

## 3 Results

### 3.1 Comparison Between Leakage Group and Non‐Leakage Group According to Patient Background Characteristics and Parameters of the Gastric Conduit

The patient background characteristics and gastric conduit parameters are summarized in Table 1. Among the 136 patients, anastomotic leakage was observed in 22 (16.2%). Various factors were compared between the leakage (22 cases) and non‐leakage (114 cases) groups. There were no significant differences between the two groups in terms of preoperative treatment, history of arterial atherosclerotic disease, preoperative blood test data, or surgical or pathological factors. There were no significant differences between the two groups in the length of the gastric conduit measured before elevation via the retrosternal route or the length of the resected distal end of the gastric conduit dissected for anastomosis after elevation via the retrosternal route. The anastomosis was performed proximal to point C in all but one patient. It was located distal to point B in 62 patients and proximal to point B in 74 patients. Distances B, C, and A from the pylorus also showed no significant differences between the two groups. However, the BA distance was significantly longer in the leakage group (*p* = 0.018), whereas the distance AC was shorter in the leakage group (*p* = 0.016). Distance BC was not significantly associated with anastomotic leakage. The presence of an arcade between the right and left gastroepiploic arteries did not differ significantly between the two groups. Figure 2 shows the distribution of anastomotic position and the corresponding incidence of anastomotic leakage according to distance BA and distance AC, respectively. This trend indicated that a more proximal anastomotic position was associated with a lower incidence of anastomotic leakage.

**TABLE 1:** Patient background characteristics and parameters of the gastric conduit.

Factors	Patients (*n* = 136)	Leakage (*n* = 22)	Non‐leakage (*n* = 114)	*p*‐value
Preoperative factors				
Age (years; mean)	66.7 ± 9.20	65.4 ± 8.70	67.0 ± 9.31	0.306
Sex				
male/female	111/25	19/3	92/22	0.765
Height (cm; mean)	163.0 ± 12.9	164.5 ± 7.30	163.2 ± 13.7	0.523
Weight (kg; mean)	59.7 ± 14.7	62.1 ± 14.0	59.2 ± 14.8	0.388
Histology				
squamous/adeno/others	113/12/11	15/3/4	98/9/7	0.068
Tumor location				
upper/middle/lower	18/75/43	2/14/6	16/61/37	0.765
Preoperative therapy				
chemotherapy/chemoradiotherapy/none	58/13/65	5/2/15	53/11/50	0.085
Arteriosclerotic disease				
yes/no	72/64	10/12	62/52	0.490
Serum albumin (g/dL; mean)	3.95 ± 0.42	4.00 ± 0.31	3.94 ± 0.44	0.633
Hemoglobin (g/dL; mean)	11.9 ± 1.75	12.5 ± 1.76	11.8 ± 1.73	0.070
Total cholesterol (mg/dL; mean)	203 ± 40.1	205 ± 38.2	203 ± 40.6	0.985
Surgical factors				
Thoracoscopic surgery				
yes/no	123/13	20/2	103/11	1.000
Preparation of gastric conduit				
HALS/laparotomy	102/34	15/7	87/27	0.430
Operation time (min; mean)	484 ± 74.3	464 ± 79.2	488 ± 73.0	0.160
Intraoperative blood loss (mL; mean)	360 ± 314	402 ± 391	352 ± 299	0.730
Pathological factors				
pT				
T1/≧ T2	65/71	15/7	50/64	0.060
pN				
N0/≧ N1	65/71	11/11	54/60	1.000
pStage				
StageI/≧ StageII	51/85	11/11	40/74	0.469
Gastric conduit				
Length of the gastric conduit (cm; mean)	34.7 ± 3.90	35.0 ± 3.35	34.6 ± 4.00	0.453
Length of the resected gastric conduit (cm; mean)	7.99 ± 2.71	7.91 ± 1.85	8.00 ± 2.85	0.394
Distance A: (From pylorus; cm; mean)	26.7 ± 3.43	27.1 ± 3.08	26.6 ± 3.50	0.394
Distance B: (From pylorus; cm; mean)	26.2 ± 4.00	25.0 ± 3.08	26.4 ± 4.13	0.199
Distance C: (From pylorus; cm; mean)	32.2 ± 3.94	31.1 ± 3.85	32.5 ± 3.94	0.235
Distance BA (cm; mean)	0.45 ± 3.22	2.05 ± 2.67	0.15 ± 3.23	0.018
Distance AC (cm; mean)	5.58 ± 3.29	4.00 ± 2.98	5.89 ± 3.27	0.016
Distance BC (cm; mean)	6.04 ± 3.61	6.05 ± 3.46	6.03 ± 3.65	0.724
Connection of gastroepiploic arteries (yes/no)	34/102	4/18	30/84	0.592
Pre‐speed (cm/s; mean)	2.88 ± 1.08	1.88 ± 0.31	3.07 ± 1.07	< 0.001

Abbreviations: HALS, hand‐assisted laparoscopic surgery; ICG, indocyanine green.

**FIGURE 2:**
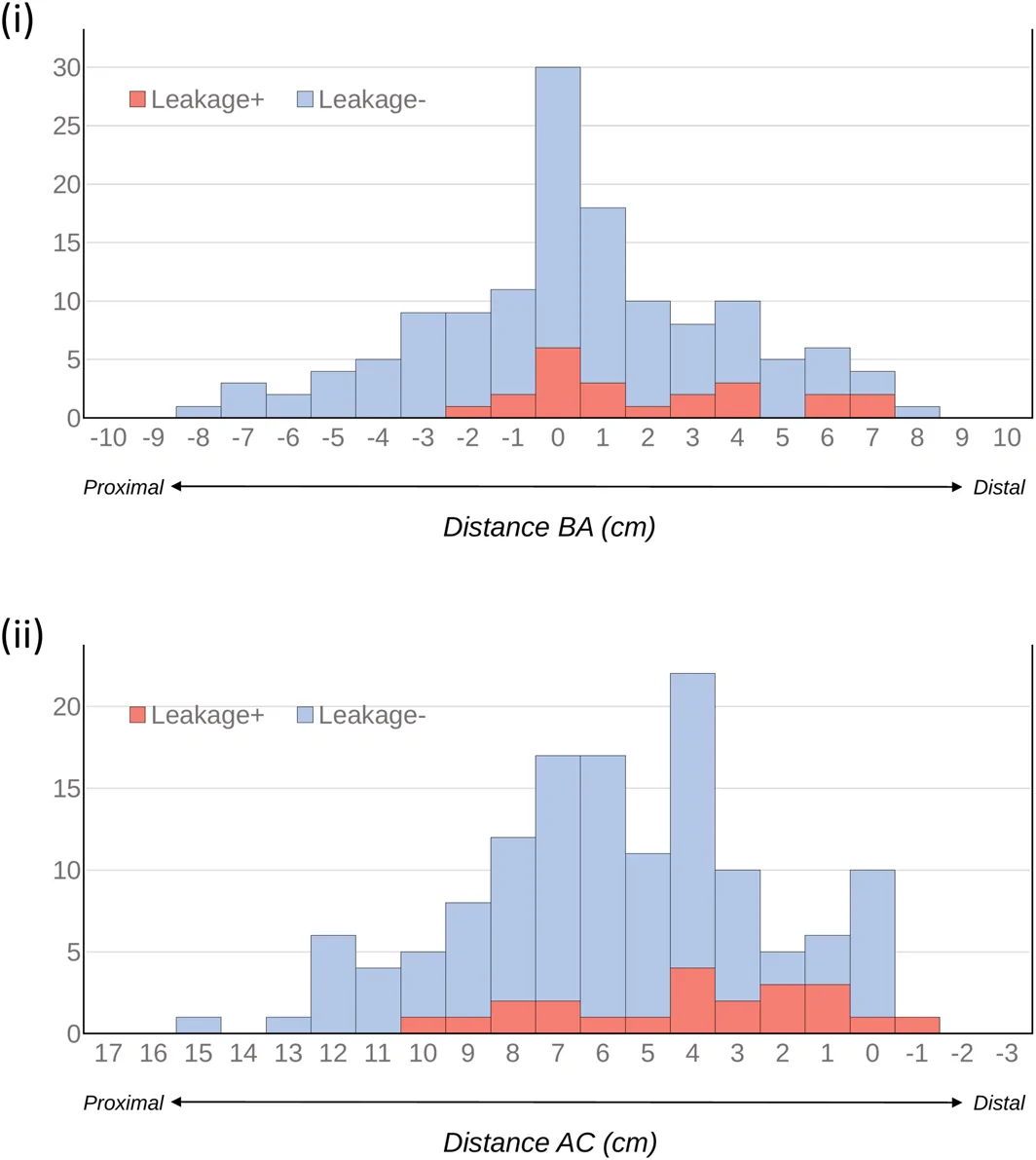
Distribution of anastomotic position and its association with anastomotic leakage. (i) Distribution of distance BA, defined as the distance from point B (the terminal end of the right gastroepiploic artery) to the anastomotic site (point A). (ii) Distribution of distance AC, defined as the distance from the anastomotic site (point A) to point C (the terminal end of the gastric conduit wall). Bars represent the number of patients at each anastomotic position; red bars indicate patients with anastomotic leakage.

### 3.2 Blood Flow Speed of the Gastric Conduit

The mean pre‐speed of the gastric conduit wall between the pylorus and point C, evaluated using ICG fluorescence imaging, was 2.88 cm/s. However, in the anastomotic leakage group, the mean pre‐speed was 1.88 cm/s, whereas in the non‐leakage group, it was 3.07 cm/s. There was a significant delay in blood flow speed in the leakage group (*p* < 0.001) (Table 1). Blood flow speed did not differ significantly between patients with and without gastroepiploic arterial communication (*p* = 0.592). We compared blood flow speed between the proximal and distal regions of the gastric conduit, using point B as the boundary. The mean proximal pre‐speed was 4.11 ± 1.60 cm/s (from the pylorus to point B) and the mean distal pre‐speed was 1.32 ± 1.37 cm/s (from point B to point C) in all patients. There was a significant delay in blood flow speed in the distal region (*p* < 0.001).

### 3.3 Risk Factor for Anastomotic Leakage

To evaluate the independent associations of the ICG‐derived parameters with anastomotic leakage, multivariable logistic regression analysis was performed. Distance BA, distance AC, and pre‐speed were included in the model. Cutoff values for these continuous variables were determined using ROC curve analysis (Figure 3). The AUC values for distance BA, distance AC, and pre‐speed were 0.659, 0.663, and 0.921, respectively. The multivariable analysis revealed that distance BA > −0.5 cm and pre‐speed < 2.19 cm/s were independent risk factors for anastomotic leakage (odds ratio [OR], 4.67; 95% confidence interval [CI], 1.03–21.20; *p* = 0.046, and OR, 56.16; 95% CI, 11.51–274.09; *p* < 0.001, respectively). Distance AC < 4.0 cm was not independently associated with anastomotic leakage (OR, 2.12; 95% CI, 0.58–7.70; *p* = 0.253) (Table 2).

**FIGURE 3:**
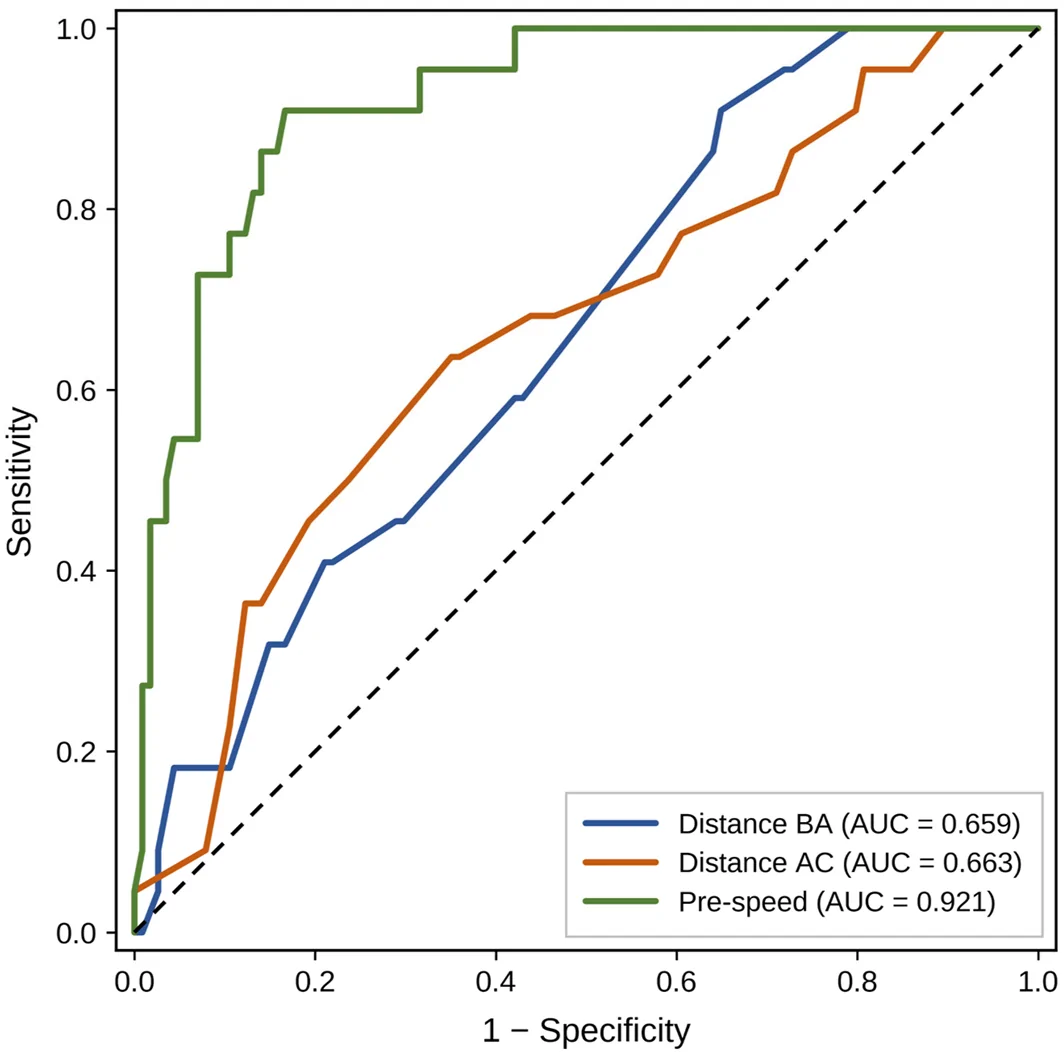
Receiver operating characteristic (ROC) curves for distance BA, distance AC, and pre‐speed in predicting anastomotic leakage.

**TABLE 2:** Multivariable analysis of risk factors for anastomotic leakage.

Factors	Odds ratio	95% CI	*p*‐value
Distance BA > −0.50 cm^a^	4.67	1.03–21.20	0.046
Distance AC < 4.00 cm^a^	2.12	0.58–7.70	0.253
Pre‐speed < 2.19 cm/s^a^	56.16	11.51–274.09	< 0.001

*Note:* Multivariable logistic regression analysis included the three predefined ICG‐derived variables: distance BA, distance AC, and pre‐speed.

Abbreviations: CI, confidence interval; ROC, receiver operating characteristic.

^a^ Individual cutoff values were determined by ROC curve analysis using the Youden index.

### 3.4 Risk Classification Based on Anastomotic Position and Pre‐Speed

Figure 4 shows scatter plots of pre‐speed versus distance BA (Figure 4A) and distance AC (Figure 4B), with their respective cutoff values for anastomotic leakage. Patients were divided into four groups according to the distance and pre‐speed cutoff values for anastomotic leakage: (i) distal/delayed group, (ii) proximal/delayed group, (iii) distal/non‐delayed group, and (iv) proximal/non‐delayed group. “Distal” refers to an anastomotic site located on the gastric conduit tip side beyond the respective cutoff value for distance BA or AC, whereas “proximal” refers to an anastomotic site located on the pylorus side relative to the cutoff value. As shown in Figure 4A, the anastomotic leakage rates in groups (i), (ii), (iii), and (iv) were 66.7%, 16.7%, 1.5%, and 3.1%, respectively. As shown in Figure 4B, the anastomotic leakage rates in groups (i), (ii), (iii), and (iv) were 66.7%, 41.7%, 4.3%, and 1.4%, respectively. The results of the pairwise comparisons of anastomotic leakage rates are shown in Table 3. The incidence of anastomotic leakage was lower in the non‐delayed group than in the delayed group. In the non‐delayed group, no significant difference in anastomotic leakage rates was observed according to anastomotic position. In contrast, in the delayed group, the anastomotic leakage rate differed significantly according to anastomotic position, with a significantly lower leakage rate in the proximal group than in the distal group (Figure 4A and Table 3; *p* = 0.034).

**FIGURE 4:**
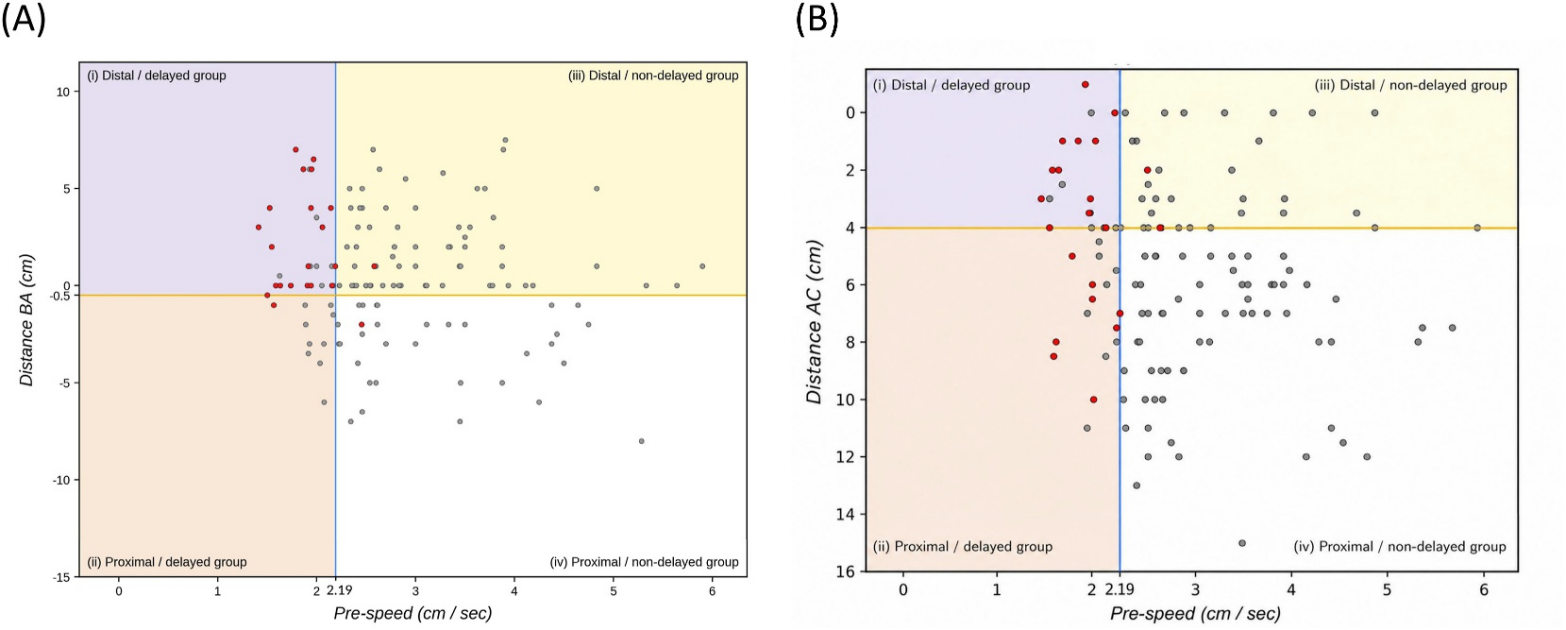
Association of pre‐speed and anastomotic position with anastomotic leakage. (A) Scatter plot of pre‐speed and distance BA. The vertical line represents the pre‐speed cutoff (2.19 cm/s), and the horizontal line represents the distance BA cutoff (−0.5 cm). (B) Scatter plot of pre‐speed and distance AC. The vertical line represents the pre‐speed cutoff (2.19 cm/s), and the horizontal line represents the distance AC cutoff (4.0 cm). Red plots indicate patients with anastomotic leakage. In both panels, patients were categorized into four groups according to blood flow speed and anastomotic position.

**TABLE 3:** Pairwise comparisons of anastomotic leakage rates according to anastomotic site and pre‐speed.

Comparison	Leakage rate	Leakage rate	Adjusted *p*‐value
Distance BA and pre‐speed classification			
(i) versus (ii)	(i) 18/27 (66.7%)	(ii) 2/12 (16.7%)	0.034
(i) versus (iii)	(i) 18/27 (66.7%)	(iii) 1/65 (1.5%)	< 0.001
(i) versus (iv)	(i) 18/27 (66.7%)	(iv) 1/32 (3.1%)	< 0.001
(ii) versus (iii)	(ii) 2/12 (16.7%)	(iii) 1/65 (1.5%)	0.370
(ii) versus (iv)	(ii) 2/12 (16.7%)	(iv) 1/32 (3.1%)	1.000
(iii) versus (iv)	(iii) 1/65 (1.5%)	(iv) 1/32 (3.1%)	1.000
Distance AC and pre‐speed classification			
(i) versus (ii)	(i) 10/15 (66.7%)	(ii) 10/24 (41.7%)	1.000
(i) versus (iii)	(i) 10/15 (66.7%)	(iii) 1/23 (4.3%)	< 0.001
(i) versus (iv)	(i) 10/15 (66.7%)	(iv) 1/74 (1.4%)	< 0.001
(ii) versus (iii)	(ii) 10/24 (41.7%)	(iii) 1/23 (4.3%)	0.026
(ii) versus (iv)	(ii) 10/24 (41.7%)	(iv) 1/74 (1.4%)	< 0.001
(iii) versus (iv)	(iii) 1/23 (4.3%)	(iv) 1/74 (1.4%)	1.000

*Note:* (i) Distal/delayed group, (ii) Proximal/delayed group, (iii) Distal/non‐delayed group, (iv) Proximal/non‐delayed group.

## 4 Discussion

In this study, we demonstrated the associations of anastomotic position and blood flow within the gastric conduit with anastomotic leakage after esophagectomy using objective ICG fluorescence measurements. Our findings suggest that both adequate blood flow and a more proximal anastomotic position may be associated with a lower risk of anastomotic leakage.

Previous studies using ICG fluorescence have demonstrated an association between gastric conduit perfusion and anastomotic leakage and emphasized the importance of performing the anastomosis at sites with better perfusion [16, 17, 18, 19, 20, 21, 22, 23]. However, no studies have specifically investigated whether the anastomotic position itself influences blood flow and subsequent leakage. The present study is the first to quantitatively assess the association between anastomotic position and anastomotic leakage using ICG fluorescence imaging.

Blood flow is generally considered to be superior on the proximal side of the gastric conduit, as the right gastroepiploic artery is its primary source of perfusion, while blood flow in the distal portion depends mainly on intramural flow. Although gastric conduit length, represented by the distances from the pylorus to points B and C, was not associated with anastomotic leakage, a more distal anastomotic position based on distance BA was independently associated with an increased risk of leakage. Distance AC showed a similar trend but was not independently associated with leakage in the multivariable analysis.

Importantly, even within areas showing positive ICG fluorescence, blood flow was significantly slower in the distal than in the proximal gastric conduit. Thus, ICG positivity alone does not guarantee sufficient perfusion for a safe anastomosis. These findings suggest that selection of a safe anastomotic site should consider not only ICG positivity but also the relative anastomotic position within the perfused area.

Consistent with our previous work, delayed blood flow speed was again identified as a strong risk factor for anastomotic leakage [10, 11, 21, 22, 23]. Because blood flow speed can be evaluated before reconstruction, identifying strategies to prevent leakage in patients with delayed flow is clinically important. As shown in Figure 4A, patients with both a distal anastomotic position and delayed blood flow had the highest leakage rate, whereas the leakage rate was significantly lower in the proximal/delayed group. These findings suggest that anastomotic position provides additional risk stratification beyond blood flow speed alone, particularly in patients with delayed flow. Accordingly, the terminal end of the right gastroepiploic artery identified by ICG fluorescence may serve as a useful anatomical landmark for determining the anastomotic position. Although AC distance provides complementary information regarding the remaining perfused gastric conduit distal to the anastomosis, its cutoff value should be interpreted cautiously because AC distance was not independently associated with anastomotic leakage in the multivariable analysis.

Our findings should not be interpreted as indicating that gastric conduit reconstruction should be abandoned whenever any single parameter exceeds its cutoff value. Rather, blood flow speed and anastomotic position should be considered together during intraoperative decision‐making. Among the anatomical parameters evaluated, BA distance may provide a more direct and clinically relevant indicator of anastomotic position, whereas AC distance may provide complementary information regarding the remaining perfused gastric conduit. Previous reports have shown that the Kocher maneuver can increase gastric conduit reach by approximately 2 cm, potentially allowing a more proximal anastomosis [24]. When anatomically feasible, such mobilization may be considered to achieve a more proximal anastomotic position. Ultimately, the reconstructive strategy should be determined comprehensively based on both perfusion and anatomical feasibility rather than on any single parameter.

This study has several limitations. First, this was a retrospective, single‐center study, and only patients who underwent retrosternal reconstruction were included. Therefore, our findings may not be directly applicable to other reconstruction routes, such as the posterior mediastinal route, where conduit compression and tension may differ. Second, several limitations were related to surgical and perfusion assessment. ICG fluorescence imaging was performed before reconstruction, and changes in conduit perfusion after pull‐up were not directly evaluated. Our previous study using the same patient cohort demonstrated significantly decreased blood flow speed after pull‐up, suggesting impaired conduit perfusion due to thoracic inlet compression [11]. In addition, although Kocher mobilization was selectively performed when additional conduit length was required, its use was not routinely recorded, precluding evaluation of its potential influence on anastomotic leakage. Third, there were limitations related to the measurement methodology. Because PDE was used for ICG fluorescence imaging, the applicability of our measurements to other imaging systems remains unclear [25]. Blood flow speed was reevaluated by a single observer, and interobserver reproducibility was not assessed. Gastroepiploic arterial communication was assessed by intraoperative macroscopic observation rather than objective angiographic or anatomical evaluation; therefore, its reported prevalence may differ from that in anatomical studies, and the subjective nature of this assessment may limit reproducibility [26].

In conclusion, this study demonstrated that ICG fluorescence imaging provides useful information for determining an appropriate anastomotic position. Selecting a more proximal anastomotic site within the gastric conduit may help reduce the risk of anastomotic leakage.

## Author Contributions

**Yamato Ninomiya:** conceptualization, data curation, formal analysis, writing – original draft. **Kazuo Koyanagi:** conceptualization, supervision, writing – review and editing. **Rie Nakashima:** data curation. **Kohei Tajima:** data curation. **Kohei Kanamori:** data curation. **Mika Ogimi:** data curation. **Yoshiaki Shoji:** data curation, writing – review and editing. **Akihito Kazuno:** data curation. **Miho Yamamoto:** data curation. **Masaki Mori:** supervision, writing – review and editing.

## Funding

The authors have nothing to report.

## Ethics Statement

This retrospective study was approved by the Institutional Review Board (Approval number: 13R‐209). The study was conducted in accordance with the Declaration of Helsinki (revised in Fortaleza, Brazil, October 2013).

## Consent

Written informed consent was obtained from all patients included in this study.

## Conflicts of Interest

The authors declare no conflicts of interest.

## Data Availability

The data that support the findings of this study are available from the corresponding author upon reasonable request. The data are not publicly available due to privacy or ethical restrictions.
